# The genome sequence of the heath fritillary, *Melitaea athalia* (Rottemburg, 1775)

**DOI:** 10.12688/wellcomeopenres.17280.1

**Published:** 2021-11-10

**Authors:** Alex Hayward, Roger Vila, Dominik R. Laetsch, Konrad Lohse, Tobias Baril, Darwin Tree of Life Barcoding collective, Wellcome Sanger Institute Tree of Life programme, Wellcome Sanger Institute Scientific Operations: DNA Pipelines collective, Tree of Life Core Informatics collective, Darwin Tree of Life Consortium

**Affiliations:** 1 University of Exeter, Penryn, UK; 2 Institut de Biologia Evolutiva (CSIC - Universitat Pompeu Fabra), Barcelona, Spain; 3 Institute of Evolutionary Biology, University of Edinburgh, Edinburgh, UK

**Keywords:** Melitaea athalia, Mellicta athalia, heath fritillary, genome sequence, chromosomal

## Abstract

We present a genome assembly from an individual female *Melitaea athalia* (also known as *Mellicta athalia*; the heath fritillary; Arthropoda; Insecta; Lepidoptera; Nymphalidae). The genome sequence is 610 megabases in span. In total, 99.98% of the assembly is scaffolded into 32 chromosomal pseudomolecules, with the W and Z sex chromosome assembled. Gene annotation of this assembly on Ensembl has identified 12,824 protein coding genes.

## Species taxonomy

Eukaryota; Metazoa; Ecdysozoa; Arthropoda; Hexapoda; Insecta; Pterygota; Neoptera; Endopterygota; Lepidoptera; Glossata; Ditrysia; Papilionoidea; Nymphalidae; Nymphalinae; *Melitaea athalia* (also known as *Mellicta athalia*) (Rottemburg, 1775) (NCBI:txid113330).

## Introduction

The heath fritillary, *Melitaea athalia* (also known as *Mellicta athalia*), is a medium-small sized butterfly found throughout the Palaearctic from western Europe to Japan. Historically, the species has been linked with the traditional practice of woodland coppicing, earning it the nickname of ‘Woodman’s Follower’. *M. athalia* is one of the UK’s rarest butterflies and was on the brink of extinction during the 1970s, but conservation efforts have since helped to save the species ( Warren, 1987). In the UK *M. athalia* is restricted to grasslands in Cornwall and Devon, heathland in Exmoor, and coppiced woodland in Kent and Essex ( Tomlinson & Still, 2002) and is a species of principal importance under the Natural Environment and Rural Communities Act 2006. However, it is listed as Least Concern in the IUCN Red List (Europe) ( van Swaay *et al.*, 2010). Up to eight forms and subspecies are recognized in Europe ( Tolman & Lewington, 1997). The taxon *celadussa* Fruhstorfer, 1910, originally described as a subspecies of *athalia* from southwestern Europe, is now recognized by many authors as a distinct parapatric species, with a contact zone extending from France to Austria where hybrids are found ( Wiemers *et al.*, 2018). Univoltine Fennoscandian and southern European alpine subspecies fly in single broods (June-July), whilst subalpine subspecies are bivoltine and fly during May-June and late July-August ( Tolman & Lewington, 1997). Females of *M. athalia* lay eggs in batches on the underside of leaves of a wide range of herbaceus food plants, with caterpillars feeding, aestivating, and hibernating together in silk nests ( Wahlberg, 2000). The standard haploid karyotype of *M. athalia* consists of 30 autosomes and one sex chromsome ( Bátori *et al.*, 2012), and the female is heterogametic (WZ).

## Genome sequence report

**Figure 1.**
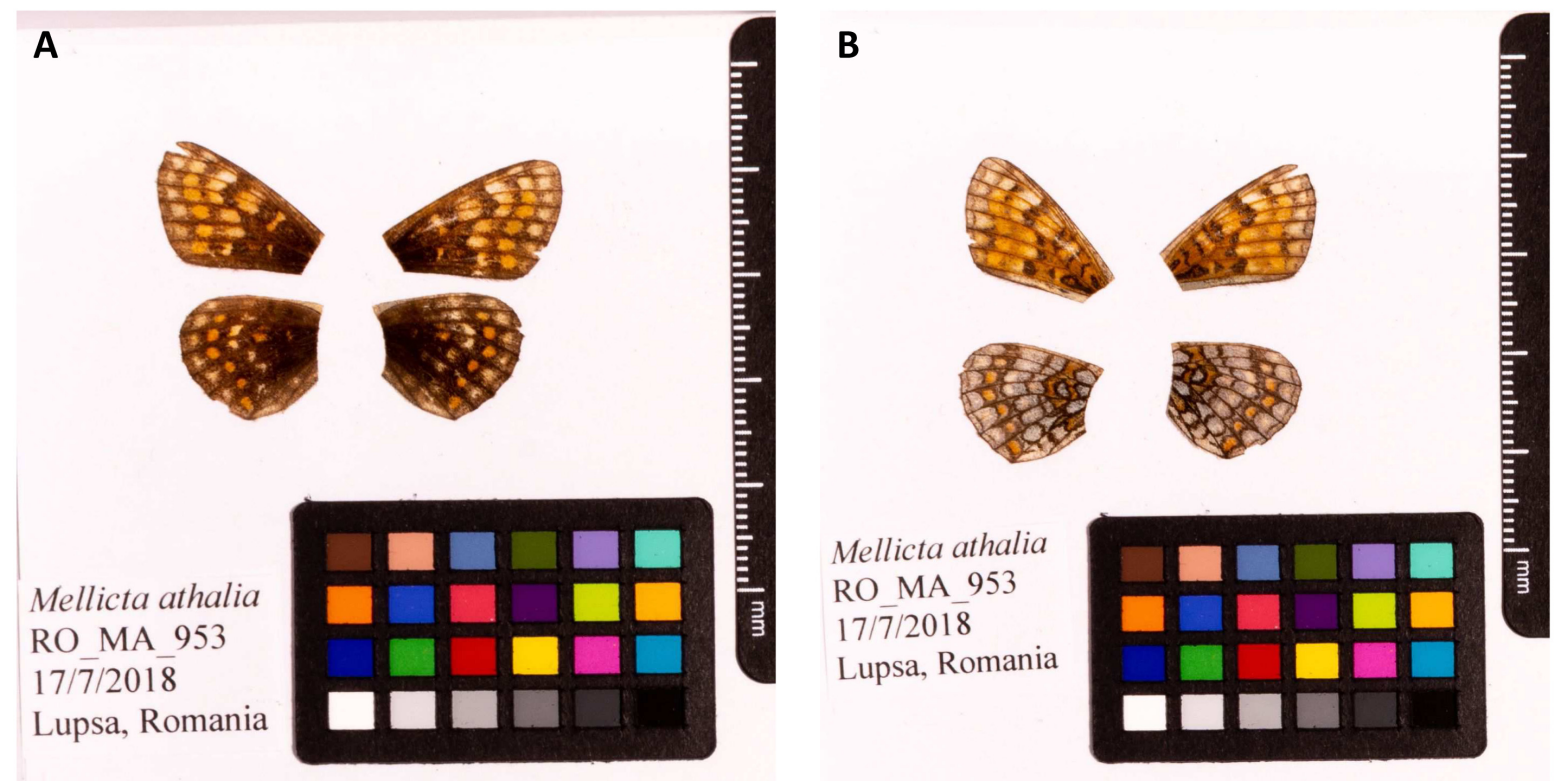
Fore and hind wings of *Melitaea athalia* specimen from which the genome was sequenced. ( **A**) Dorsal surface view of wings from specimen RO_MA_953 (ilMelAtha1.1) from Lupşa, Transylvania, Romania, used to generate Pacific Biosciences and 10X genomics data. ( **B**) Ventral surface view of wings from specimen RO_MA_953 (ilMelAtha1.1) from Lupşa, Transylvania, Romania, used to generate Pacific Biosciences and 10X genomics data.

The genome was sequenced from a single female *M. athalia* collected from Lupşa, Transylvania, Romania (latitude 46.416, longitude 23.192) ( Figure 1). A total of 30-fold coverage in Pacific Biosciences single-molecule long reads (N50 16 kb) and 64-fold coverage in 10X Genomics read clouds were generated. Primary assembly contigs were scaffolded with chromosome conformation Hi-C data. Manual assembly curation corrected 82 missing/misjoins and removed 19 haplotypic duplications, reducing the assembly size by 1.94% and scaffold number by 45.12%, and increasing the scaffold N50 by 7.20%.

**Figure 2.**
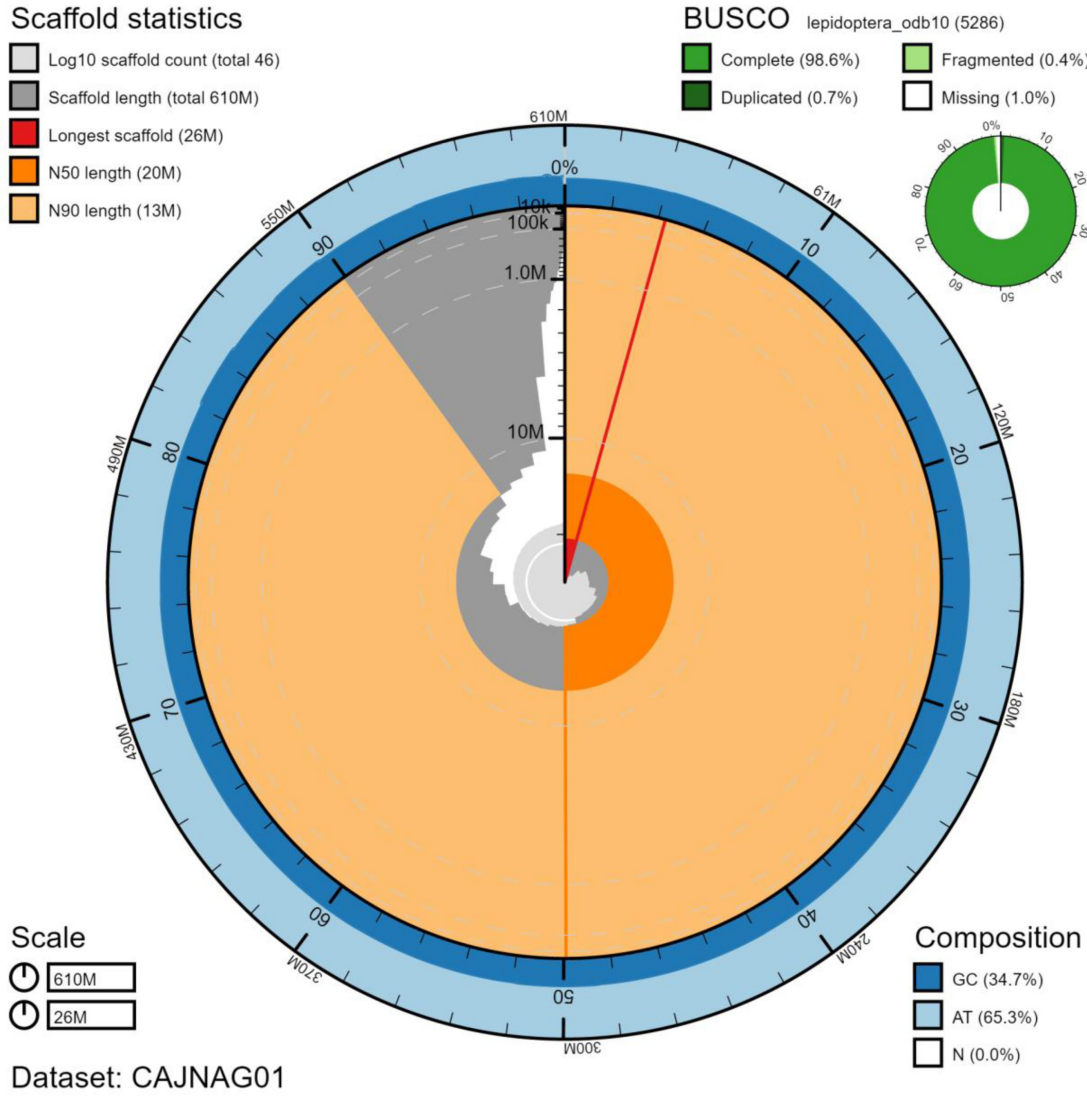
Genome assembly of *Mellitaea athalia*, ilMelAtha1.1: metrics. The BlobToolKit Snailplot shows N50 metrics and BUSCO gene completeness. The main plot is divided into 1,000 size-ordered bins around the circumference with each bin representing 0.1% of the 609,564,789 bp assembly. The distribution of scaffold lengths is shown in dark grey with the plot radius scaled to the longest chromosome present in the assembly (26,233,870 bp, shown in red). Orange and pale-orange arcs show the N50 and N90 scaffold lengths (20,295,254 and 13,271,753 bp), respectively. The pale grey spiral shows the cumulative scaffold count on a log scale with white scale lines showing successive orders of magnitude. The blue and pale-blue area around the outside of the plot shows the distribution of GC, AT and N percentages in the same bins as the inner plot. A summary of complete, fragmented, duplicated and missing BUSCO genes in the lepidoptera_odb10 set is shown in the top right. An interactive version of this figure is available at https://blobtoolkit.genomehubs.org/view/ilMelAtha1.1/dataset/CAJNAG01/snail.

The final assembly has a total length of 610 Mb in 46 sequence scaffolds with a scaffold N50 of 20 Mb ( Table 1). Of the assembly sequence, 99.98% was assigned to 32 chromosomal-level scaffolds, representing 30 autosomes (numbered by sequence length), and the W and Z sex chromosome ( Figure 2– Figure 5; Table 2). The assembly has a BUSCO ( Simão *et al.*, 2015) completeness of 98.6% (single 97.9%, duplicated 0.7%, fragmented 0.4%, missing 1.0%) using the lepidoptera_odb10 reference set. While not fully phased, the assembly deposited is of one haplotype. Contigs corresponding to the second haplotype have also been deposited.

**Figure 3.**
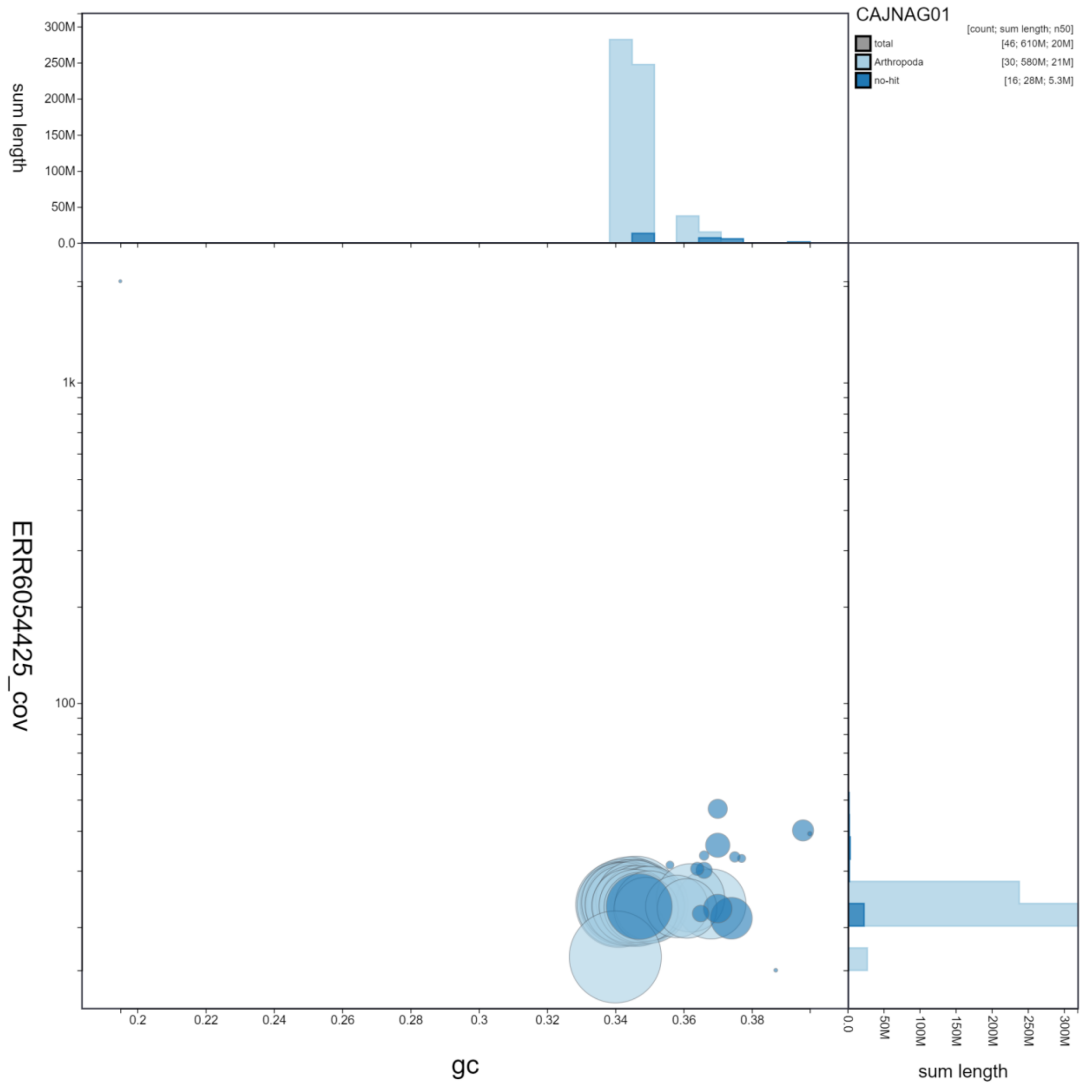
Genome assembly of *Mellitaea athalia* , ilMelAtha1.1: GC coverage. BlobToolKit GC-coverage plot. Scaffolds are coloured by phylum. Circles are sized in proportion to scaffold length. Histograms show the distribution of scaffold length sum along each axis. An interactive version of this figure is available at https://blobtoolkit.genomehubs.org/view/ilMelAtha1.1/dataset/CAJNAG01/blob.

**Figure 4.**
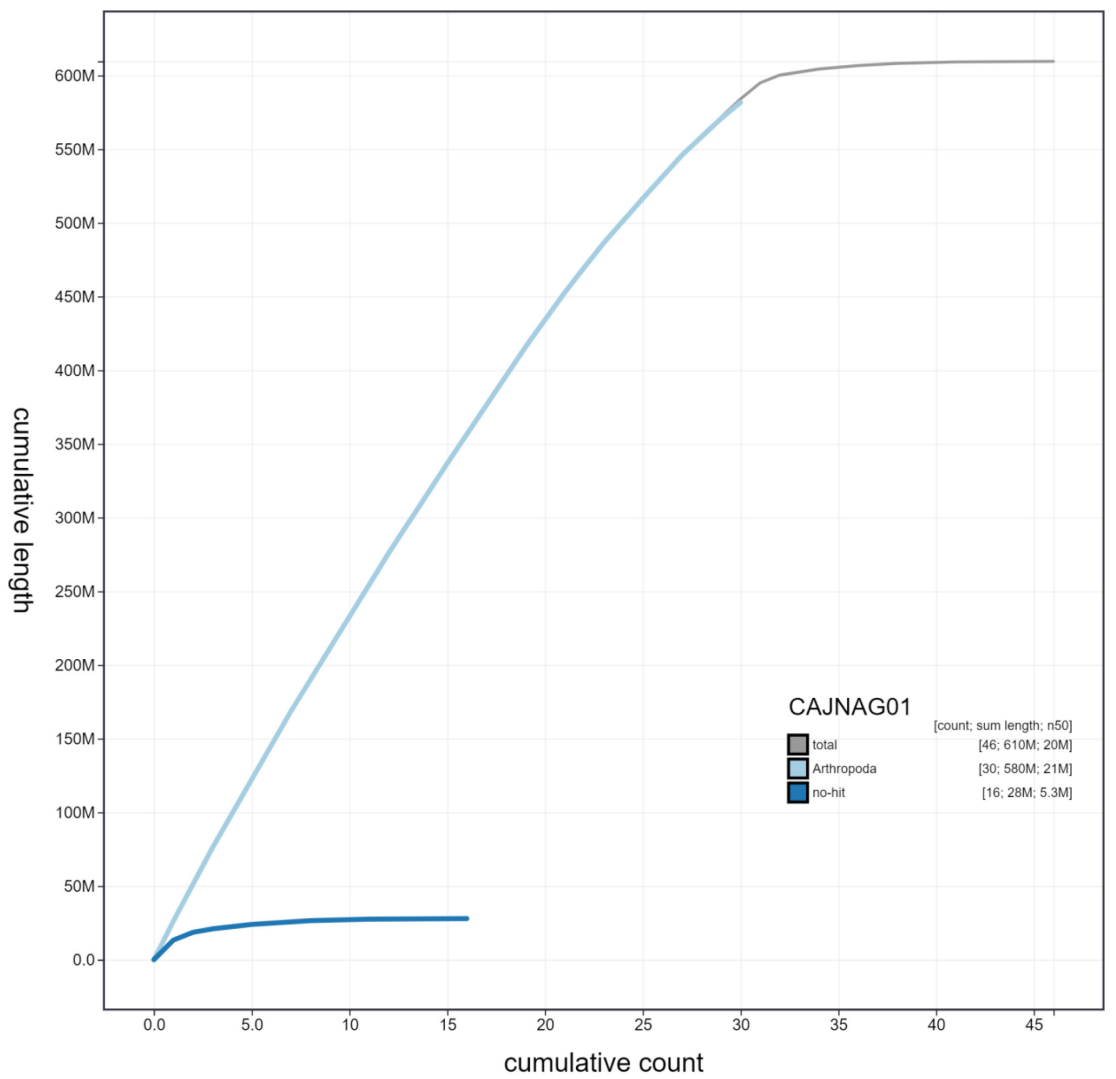
Genome assembly of *Mellitaea athalia*, ilMelAtha1.1: cumulative sequence. BlobToolKit cumulative sequence plot. The grey line shows cumulative length for all scaffolds. Coloured lines show cumulative lengths of scaffolds assigned to each phylum using the buscogenes taxrule. An interactive version of this figure is available at https://blobtoolkit.genomehubs.org/view/ilMelAtha1.1/dataset/CAJNAG01/cumulative.

**Figure 5.**
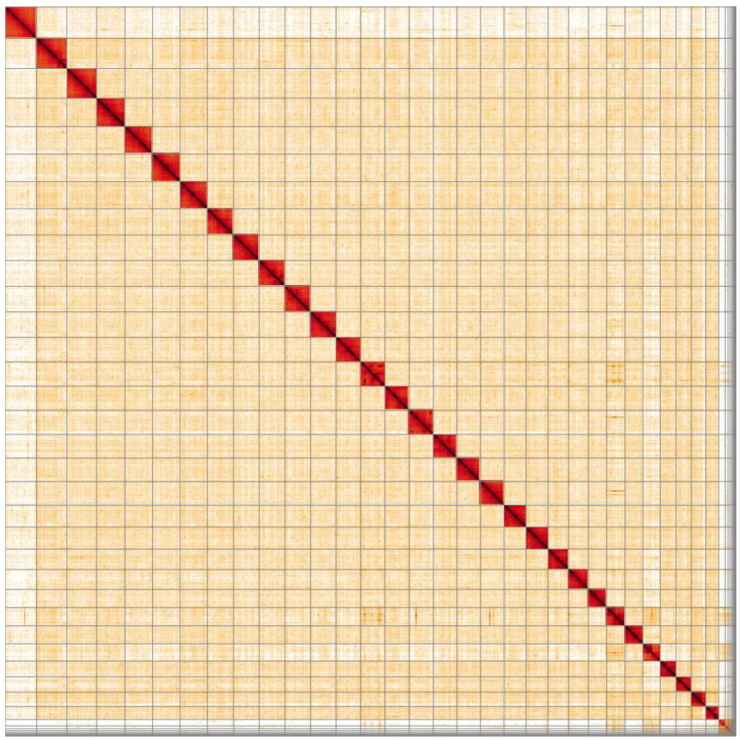
Genome assembly of *Mellitaea athalia*, ilMelAtha1.1: Hi-C contact map. Hi-C contact map of the ilMelAtha1.1 assembly, visualised in HiGlass. Chromosomes are arranged in size order from left to right and top to bottom.

**Table 1.** Genome data for *Melitaea athalia*, ilMelAtha1.1.

*Project accession data*	
Assembly identifier	ilMelAtha1.1
Species	*Melitaea athalia* (also known as *Mellicta athalia*)
Specimen	ilMelAtha1, RO_MA_953
NCBI taxonomy ID	NCBI:txid113330
BioProject	PRJEB42954
BioSample ID	SAMEA7523312
Isolate information	Female, whole organism
*Raw data accessions*	
PacificBiosciences SEQUEL II	ERR6576319
10X Genomics Illumina	ERR6054423-ERR6054426
Hi-C Illumina	ERR6054427
*Genome assembly*	
Assembly accession	GCA_905163435.1
*Accession of alternate haplotype*	GCA_905163405.1
Span (Mb)	576
Number of contigs	70
Contig N50 length (Mb)	18
Number of scaffolds	43
Scaffold N50 length (Mb)	19
Longest scaffold (Mb)	23
BUSCO * genome score	C:98.6%[S:97.9%,D:0.7%],F:0.4%,M:1.0%,n:5286
*Gene annotation*	
Number of protein coding genes	12,824
Average coding sequence length (bp)	1,492
Average number of exons per transcript	8
Average exon size (bp)	264
Average intron size (bp)	2,892

*BUSCO scores based on the lepidoptera_odb10 BUSCO set using v5.1.2. C= complete [S= single copy, D=duplicated], F=fragmented, M=missing, n=number of orthologues in comparison. A full set of BUSCO scores is available at https://blobtoolkit.genomehubs.org/view/ilMelAtha1.1/dataset/CAJNAG01/busco.

**Table 2.** Chromosomal pseudomolecules in the genome assembly of *Melitaea athalia*, ilMelAtha1.1.

INSDC accession	Chromosome	Size (Mb)	GC%
HG992177.1	1	25.13	34.6
HG992178.1	2	24.88	34.4
HG992179.1	3	23.65	34.6
HG992180.1	4	22.93	34.2
HG992181.1	5	22.90	34.1
HG992182.1	6	22.79	34.5
HG992183.1	7	21.87	34.4
HG992184.1	8	21.42	34.3
HG992185.1	9	21.39	34.2
HG992186.1	10	21.38	34.1
HG992187.1	11	21.23	34.4
HG992188.1	12	20.51	34.3
HG992189.1	13	20.30	34.8
HG992190.1	14	20.21	34.2
HG992191.1	15	19.99	34.3
HG992192.1	16	19.82	34.6
HG992193.1	17	19.64	34.5
HG992194.1	18	19.55	34.7
HG992195.1	19	18.44	34.9
HG992196.1	20	18.37	35
HG992197.1	21	17.06	34.4
HG992198.1	22	16.62	34.6
HG992199.1	23	15.22	34.7
HG992200.1	24	15.15	36.8
HG992201.1	25	14.97	35
HG992202.1	26	14.40	36.2
HG992203.1	27	13.27	34.7
HG992204.1	28	12.76	34.9
HG992205.1	29	11.97	35.8
HG992206.1	30	10.90	36.1
HG992207.1	W	5.27	37.4
HG992176.1	Z	26.23	34
HG992208.1	MT	0.02	19.7
-	Unplaced	9.34	37.2

## Gene annotation

The Ensembl gene annotation system ( Aken *et al.*, 2016) was used to generate annotation for the *Melitaea athalia* assembly (GCA_905220545.1, see https://rapid.ensembl.org/Mellicta_athalia_GCA_905220545.1/; Table 1). The annotation was created primarily through alignment of transcriptomic data to the genome, with gap filling via protein-to-genome alignments of a select set of proteins from UniProt ( UniProt Consortium, 2019)) and OrthoDB ( Kriventseva *et al.*, 2008). Prediction tools, CPC2 ( Kang *et al.*, 2017) and RNAsamba ( Camargo *et al.*, 2020), were used to aid determination of protein coding genes.

## Methods

### Sample acquisition, nucleic acid extraction and sequencing

A single female *M. athalia* was collected from Lupşa, Transylvania, Romania (latitude 46.416, longitude 23.192) by Alex Hayward (University of Exeter), Roger Vila (Universitat Pompeu Fabra), Dominik Laetsch and Konrad Lohse (both University of Edinburgh), using a net. The specimen was identified by Roger Vila and was snap-frozen in liquid nitrogen.

DNA was extracted from the whole organism of ilMelAtha1 using the Qiagen MagAttract HMW DNA kit, according to the manufacturer’s instructions. Pacific Biosciences HiFi circular consensus and 10X Genomics read cloud sequencing libraries were then constructed according to the manufacturers’ instructions. Sequencing was performed by the Scientific Operations core at the Wellcome Sanger Institute on Pacific Biosciences SEQUEL II and Illumina HiSeq X instruments. Hi-C data were generated using the Arima v1.0 kit and sequenced on HiSeq X.

### Genome assembly

Assembly was carried out with HiCanu ( Nurk *et al.*, 2020). Haplotypic duplication was identified and removed with purge_dups ( Guan *et al.*, 2020). One round of polishing was performed by aligning 10X Genomics read data to the assembly with longranger align, calling variants with freebayes ( Garrison & Marth, 2012). The assembly was then scaffolded with Hi-C data ( Rao *et al.*, 2014) using SALSA2 ( Ghurye *et al.*, 2019). The assembly was checked for contamination and corrected using the gEVAL system ( Chow *et al.*, 2016) as described previously ( Howe *et al.*, 2021). Manual curation was performed using gEVAL, HiGlass ( Kerpedjiev *et al.*, 2018) and Pretext. The mitochondrial genome was assembled using MitoHiFi ( Uliano-Silva *et al.*, 2021). The genome was analysed and BUSCO scores generated within the BlobToolKit environment ( Challis *et al.*, 2020). Table 3 contains a list of all software tool versions used, where appropriate.

**Table 3.** Software tools used.

Software tool	Version	Source
HiCanu	2.1	Nurk *et al.*, 2020
purge_dups	1.2.3	Guan *et al.*, 2020
SALSA2	2.2	Ghurye *et al.*, 2019
longranger align	2.2.2	https://support.10xgenomics.com/genome-exome/software/pipelines/latest/advanced/other-pipelines
freebayes	1.3.1-17-gaa2ace8	Garrison & Marth, 2012
MitoHiFi	1	https://github.com/marcelauliano/MitoHiFi
gEVAL	2016	Chow *et al.*, 2016
HiGlass	1.11.6	Kerpedjiev *et al.*, 2018
PretextView	0.1.x	https://github.com/wtsi-hpag/PretextView
BlobToolKit	2.6.2	Challis *et al.*, 2020

### Ethical/compliance issues

The materials that have contributed to this genome note were supplied by a Tree of Life collaborator. The Wellcome Sanger Institute employs a process whereby due diligence is carried out proportionate to the nature of the materials themselves, and the circumstances under which they have been/are to be collected and provided for use. The purpose of this is to address and mitigate any potential legal and/or ethical implications of receipt and use of the materials as part of the research project, and to ensure that in doing so we align with best practice wherever possible.

The overarching areas of consideration are:

- Ethical review of provenance and sourcing of the material;
- Legality of collection, transfer and use (national and international).

Each transfer of samples is undertaken according to a Research Collaboration Agreement or Material Transfer Agreement entered into by the Tree of Life collaborator, Genome Research Limited (operating as the Wellcome Sanger Institute) and in some circumstances other Tree of Life collaborators.

## Data availability

European Nucleotide Archive: Mellicta athalia (heath fritillary). Accession number PRJEB42954; https://identifiers.org/ena.embl/PRJEB42954.

The genome sequence is released openly for reuse. The *M. athalia* genome sequencing initiative is part of the Darwin Tree of Life (DToL) project. All raw sequence data and the assembly have been deposited in INSDC databases. Raw data and assembly accession identifiers are reported in Table 1.
