# Supplementary material for: The genome sequence of the heath fritillary, Melitaea athalia (Rottemburg, 1775)
Source: Wellcome Open Res. 2021 Nov 10;6:304. [Version 1] doi: 10.12688/wellcomeopenres.17280.1 (PMC8796007; doi:10.12688/wellcomeopenres.17280.1)
Supplement: Peer review history

# Peer review: Reviewer response for version 1

**DOI:** 10.21956/wellcomeopenres.19104.r47141

**Published:** 2022-01-27

**Authors:** Hikmet Budak

**Affiliations:** 1 Department of Genomics and Genome Editing, Montana BioAgriculture Inc., Missoula, MT, USA

I believe that there is data which contributes to the community. Both the genome sequencing and assembly  methods and applications looks good and presented well although it would be great to go into details a little more so that other scientists can use/compare/benefit form their experiences. I am unsure if Figure 4 is necessary to show cumulative seq. The authors were even able to assemble the W chromosome. This would definitely help sequencing and chromosome level assembly of wheat stem sawfly and orange wheat blossom midge, dangerous insects in the USA.

Are sufficient details of methods and materials provided to allow replication by others?

Yes

Is the rationale for creating the dataset(s) clearly described?

Yes

Are the datasets clearly presented in a useable and accessible format?

Yes

Are the protocols appropriate and is the work technically sound?

Yes

Reviewer Expertise:

Plant genomics and biology, Next generation sequencing and annotations, smallRNAs, microRNAs, LncRNAs.

I confirm that I have read this submission and believe that I have an appropriate level of expertise to confirm that it is of an acceptable scientific standard.

# Peer review: Reviewer response for version 1

**DOI:** 10.21956/wellcomeopenres.19104.r47139

**Published:** 2021-11-25

**Authors:** Brian A. Counterman

**Affiliations:** 1 Department of Biological Sciences, Auburn University, Auburn, AL, USA

This manuscript presents a concise report of the genome sequencing and assembly of *Melitaea athalia*, one of the rarest butterflies in Europe. The methods used are appropriate and appear to have been conducted appropriately. The result is a highly contiguous, chromosome-level assembly of the genome. It is noteworthy that the authors were able to assemble the W chromosome and release the unphased assemblies of both diploid copies. This genome is certain to be a valuable resource for comparative genomics of Lepidoptera and future conservation efforts of the species.

 I found no major errors or concerns with the manuscript, and felt the tables and figures included sufficiently and succinctly presented relevant information to assess the methods used and quality of the genome assembly. I was unclear which version of BUSCO was used and if the "lepidoptera_odb10" database was the 2019 version. These details could be useful for future users of the data, and perhaps could be simply added to Table 3. Overall, I commend the authors for their clear and concise presentation of the work.

Are sufficient details of methods and materials provided to allow replication by others?

Yes

Is the rationale for creating the dataset(s) clearly described?

Yes

Are the datasets clearly presented in a useable and accessible format?

Yes

Are the protocols appropriate and is the work technically sound?

Yes

Reviewer Expertise:

Lepidoptera, Evolution, Genomics

I confirm that I have read this submission and believe that I have an appropriate level of expertise to confirm that it is of an acceptable scientific standard.
